# Anti-3-Hydroxy-3-Methylglutaryl-Coenzyme A (HMG-CoA) Reductase Negative Statin-Induced Necrotizing Myopathy: A Challenging Case

**DOI:** 10.7759/cureus.105135

**Published:** 2026-03-12

**Authors:** Alexander S Kaiteris, Rahul Tripathi, George Ayob, Brandon Ng

**Affiliations:** 1 Internal Medicine, Stony Brook University Hospital, Stony Brook, USA

**Keywords:** acute kidney injury, acute myositis, muscle injury, rhabdomyolysis, statin-associated autoimmune myopathy

## Abstract

We present a case of a man in his early 70s who presented to the emergency department with two weeks of diffuse muscle weakness and dark-colored urine. His history of present illness was negative for obvious inciting events based on the admission history and physical exam. Laboratory tests in the emergency department (ED) showed evidence of end-organ dysfunction, including elevated liver enzymes with aspartate aminotransferase (AST) and alanine aminotransferase (ALT) levels of 1,197 and 1,718 IU/L, respectively, an elevated creatinine (Cr) level of 1.44 mg/dL, and significant muscle injury indicated by a creatine phosphokinase (CPK) level of 20,000 IU/L. An unspecified myopathy was high on the differential. Workup was negative for autoimmune or rheumatologic causes of myositis, and statin-induced necrotizing myopathy became the top differential. The patient was treated with pulse-dose steroids. His hospital course was complicated by acute kidney failure requiring dialysis, with eventual recovery of kidney function and normalization of clinical and laboratory findings.

## Introduction

Inflammatory myopathies have a prevalence of 2 to 33 people out of a population of 100,000. A subset of inflammatory myopathies, immune-mediated necrotizing myopathies (IMNMs), accounts for only a small proportion of cases [1]. Statin-induced necrotizing myopathy is a specific form of IMNM that has gained increasing recognition, given the widespread use of statin medications. It is a rare, debilitating form of IMNM that affects approximately two per 100,000 to two per million people [1,2]. The use of statin medications has tripled since 2008-2009, from 31 million to 92 million users, and statins are considered among the safest, most affordable, and most efficacious medications available today [3]. Typical side effects, such as myalgias or a transient rise in liver function tests (LFTs), are usually easily resolved by changing the statin medication or changing the class of medication. Statins have other, less common toxicities, such as intolerable myalgias associated with mild CPK elevation (less than four times the upper limit of normal), which occur in approximately 0.2-2 per 1,000 people. Although less common than mild myalgias, intolerable myalgias are still considered to be a benign side effect that is quickly resolved with discontinuation of the statin [4]. While statin-related myalgias are relatively benign, statin-induced necrotizing myopathy is an extremely rare and difficult-to-identify condition that may develop rapidly with minimal to no preceding symptoms, facilitating prompt diagnosis and treatment of this condition to avoid fatal outcomes. The condition most commonly presents with progressive muscle weakness involving the posterior and medial compartments of the thigh and the gluteal muscles, in association with a CPK elevation of 1,000 to 10,000 units per liter [5]. In this report, we present a unique case of presumed statin-induced necrotizing myositis in the setting of high-intensity statin use.

## Case presentation

The patient was a man in his early 70s with a past medical history significant for coronary artery disease status-post four-vessel percutaneous coronary intervention, hypertension, and hyperlipidemia who presented to the emergency department (ED) with a two-week history of dark urine and diffuse muscle weakness. The patient reported that the weakness was progressive, developing rapidly over two weeks. He denied recent sick contacts, prior episodes of acute-onset muscle weakness, trauma to the lower extremity, or injuries to the back region. He also denied starting new medications before the onset of weakness. There was no documentation or reported history of chronic back pain or spinal stenosis. He reported frequent hiking and endorsed a recent insect bite to the gluteal region, which he assumed was a tick bite and which he associated with the onset of weakness. He denied a history of gait instability, frequent falls, or a prior need for outpatient physical therapy. The patient confirms he was able to complete his activities of daily living without issue. He denied overt hematuria, but stated the color of his urine was consistent with a dark-yellow color, which was abnormal for him. Medication reconciliation was performed and confirmed the patient’s home medications, which included atorvastatin 80 mg, amlodipine 5 mg, irbesartan 300 mg, metoprolol 50 mg, and aspirin 81 mg. He reported taking a statin at the same dose several years earlier without any recent medication changes.

**Figure 1:**
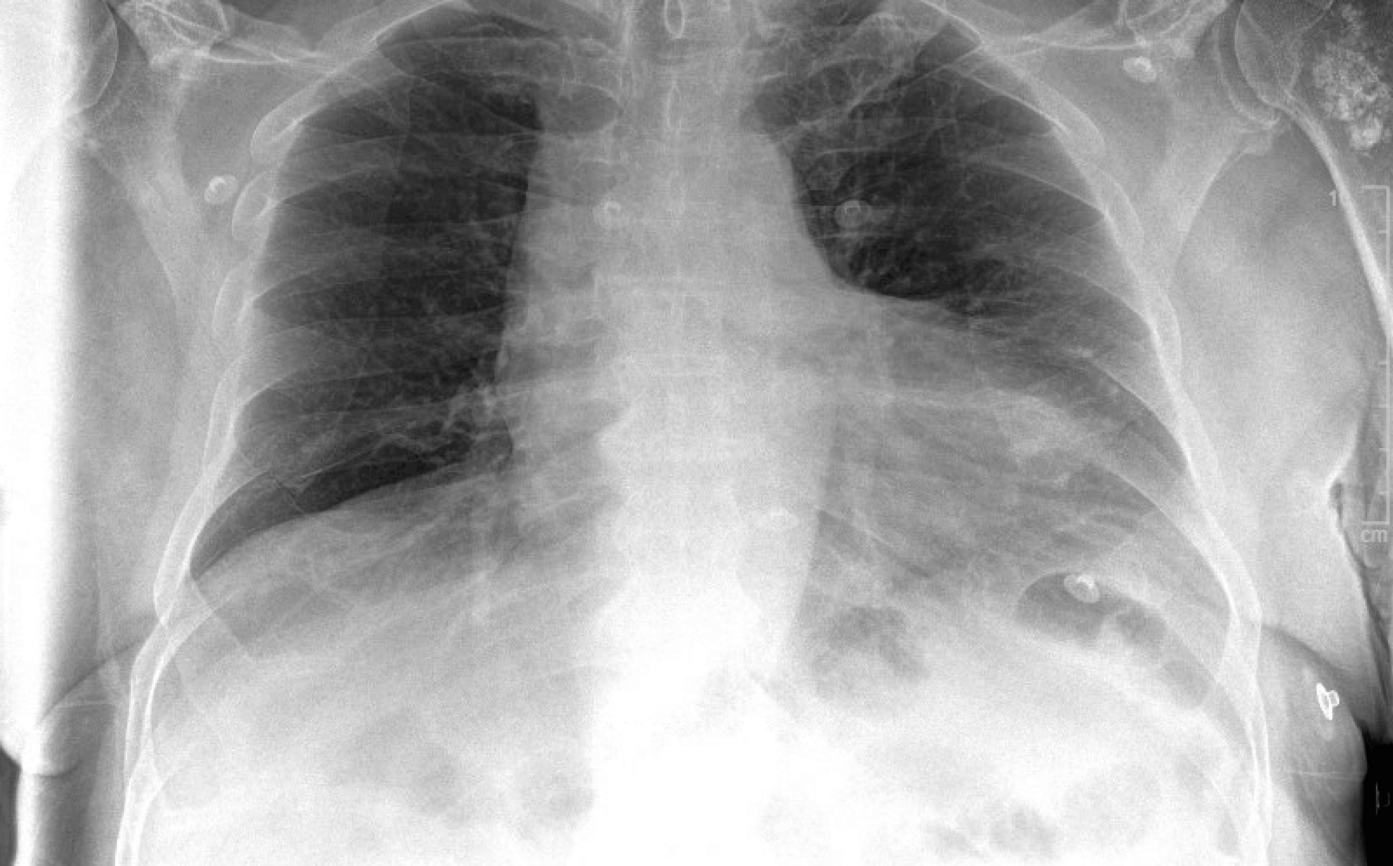
Chest X-ray showing no acute cardiopulmonary process; the cardiomediastinal silhouette is unchanged compared to the prior study.

**Figure 2:**
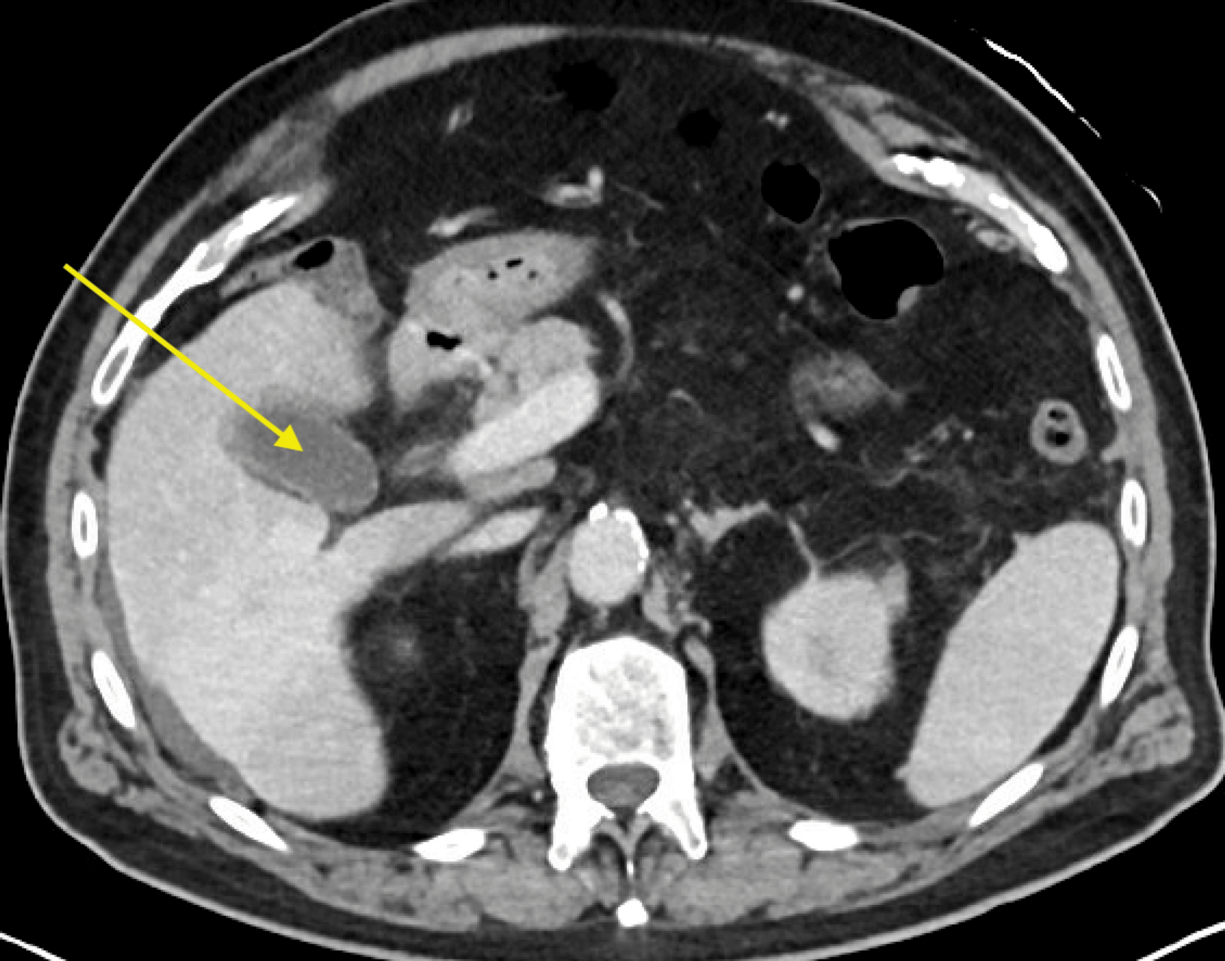
CT abdomen and pelvis showing cholelithiasis with gallbladder wall thickening and pericholecystic fluid. The gallbladder is indicated by the yellow arrow.

On arrival to the ED, vital signs were significant for a temperature of 36.7 degrees Celsius, blood pressure of 118/68 mmHg, heart rate 82 beats per minute, and oxygen saturation 98% on room air. Laboratory evaluation demonstrated elevated serum creatinine, severe transaminitis, elevated total and direct bilirubin levels, and an elevated creatine kinase level. Complete laboratory values with reference ranges are documented in Table 1. Serum electrolytes (sodium, potassium, chloride, magnesium, and phosphorus) and complete blood count panel were within normal limits. Toxicology was negative for acetaminophen, salicylates, and ethanol, and the urine drug screen was unremarkable. Chest X-ray (CXR) revealed no acute cardiopulmonary process (Figure 1). Computerized tomography (CT) of his abdomen and pelvis indicated pericholecystic fluid around the gallbladder fundus with diffuse gallbladder wall thickening (Figure 2). Social history was positive for a 30-pack-year smoking history, and he noted he had quit smoking approximately 20 years ago. He reported infrequent social alcohol consumption and denied other substance use. Two liters of intravenous fluids were administered in the ED, and he was successively admitted to the general medical service. 

**Table 1:** Laboratory values. Na, sodium; K, potassium; HCO_3_, bicarbonate; Cr, creatinine; GFR, estimated glomerular filtration rate; WBC, white blood cell count; T Bili, total bilirubin; D Bili, direct bilirubin; ALT, alanine aminotransferase; AST, aspartate aminotransferase; CPK, creatine phosphokinase; ESR, erythrocyte sedimentation rate; CRP, C-reactive protein

Lab test	Lab value (Admission)	Lab value (Hospital Day 7)	Reference range
Na	137	137	135-146 mmol/L
K	4.5	5.7	3.5-5.1 mmol/L
HCO_3_	25	16	21-31 mmol/L
Cr	1.44	4.11	0.5-1.2 mg/dL
GFR	52	15	> 60
WBC	10.9	25.3	4.8-10.8 K/uL
T Bili	3.5	2.2	0-1.2 mg/dL
D Bili	2.2	1.3	0-0.3 mg/dL
ALT	1,197	963	0-41 IU/L
AST	1,718	1,681	0-40 IU/L
CPK	21,392	>22,000	26-174 IU/L
ESR	26	31	0-20 mm/hour
CRP	1.7	2.3	0-0.5 mg/dL

The primary diagnosis on admission was rhabdomyolysis, and the patient was immediately started on continuous sodium chloride intravenous solution at a rate of 250 mL/hour. Empiric antibiotics were started with piperacillin-tazobactam out of concern for acute cholecystitis. General surgery was consulted and recommended a delayed cholecystectomy. Gastroenterology was also consulted as recommended, and magnetic resonance cholangiopancreatography (MRCP) was performed, showing hyperbilirubinemia and profound transaminitis with concern for biliary duct pathology. MRCP demonstrated no acute biliary processes. Atorvastatin was held on admission, given both rhabdomyolysis and severe transaminitis.

Given there was no clear culprit for the current presentation, an expanded laboratory workup was ordered, which included infectious, rheumatologic, and vasculitic studies (Table 2). Nucleic acid amplification testing for cytomegalovirus and herpes simplex virus was negative. Serologic testing for Ehrlichia and Anaplasma, as well as an extended myositis panel were negative. Hepatitis serology was negative. Inflammatory markers, including C-reactive protein (CRP) and erythrocyte sedimentation rate (ESR), were positive, and procalcitonin returned negative. Lyme disease testing for VlsE1 IgM/IgG yielded a low-positive result (Table 2). Urinalysis was positive for large amounts of blood with only three red blood cells, and 100 mg/dL of protein, with no evidence of urinary tract infection.

**Table 2:** Serologic testing. Lyme VlsE1/pepC10 IgM/IgG, Lyme disease serology; anti-dsDNA, anti–double-stranded DNA antibody; anti-ENA Ro (SSA), anti-Ro extractable nuclear antigen antibody; anti-ENA La (SSB), anti-La extractable nuclear antigen antibody; anti-ENA Sm, anti-Smith extractable nuclear antigen antibody; anti-ENA RNP, anti-ribonucleoprotein extractable nuclear antigen antibody; SM/RNP, combined anti-Smith/anti-RNP antibodies; chromatin Ab, anti-chromatin antibody; SCL-70 IgG, anti–topoisomerase I antibody; JO-1, anti–histidyl-tRNA synthetase antibody; centromere B Ab, anti-centromere B antibody; MPO IgG, anti-myeloperoxidase antibody; PR-3 IgG, anti-proteinase 3 antibody; GBM Ab, anti–glomerular basement membrane antibody; Babesia microti IgG/IgM, Babesia microti antibodies; EBV VCA IgG/IgM, Epstein-Barr virus viral capsid antigen antibodies; anti-PM/Scl-75, polymyositis/scleroderma-75 antibody

Lab test	Laboratory findings during hospital course	Reference range
Lyme VlsE1/pepC10 IgM/IgG Index value	1.32	<0.91
Anti-dsDNA	<1	<5 IU/mL
Anti-ENA Ro	0.8	<1.0 AI
Anti-ENA La	<0.2	<1.0 AI
Anti-ENA Sm	0.2	<1.0 AI
Anti-ENA RNP	<0.2	<1.0 AI
SM/RNP combined Ab	0.2	<1.0 AI
Chromatin Ab	2.1	<1.0 AI
SCL-70 IgG Ab	<0.2	<1.0 AI
JO-1 Ab	<0.2	<1.0 AI
Centromere B Ab	<0.2	<1.0 AI
MPO IgG Ab	<0.2	<1.0 AI
PR-3 IgG Ab	<0.2	<1.0 AI
Glomerular Basement Membrane Ab	<0.2	<1.0 AI
Babesia microti IgG Ab	<1:16	<1:16
Babesia microti IgM Ab	<1:20	<1:20
EBV VCA IgG	709.0 (High)	0.0-21.9 U/mL
EBV VCA IgM	<10.0	0.0-43.9 U/mL
Anti-PM/Scl-75 Ab (RDL)	<20	<20

**Figure 3:**
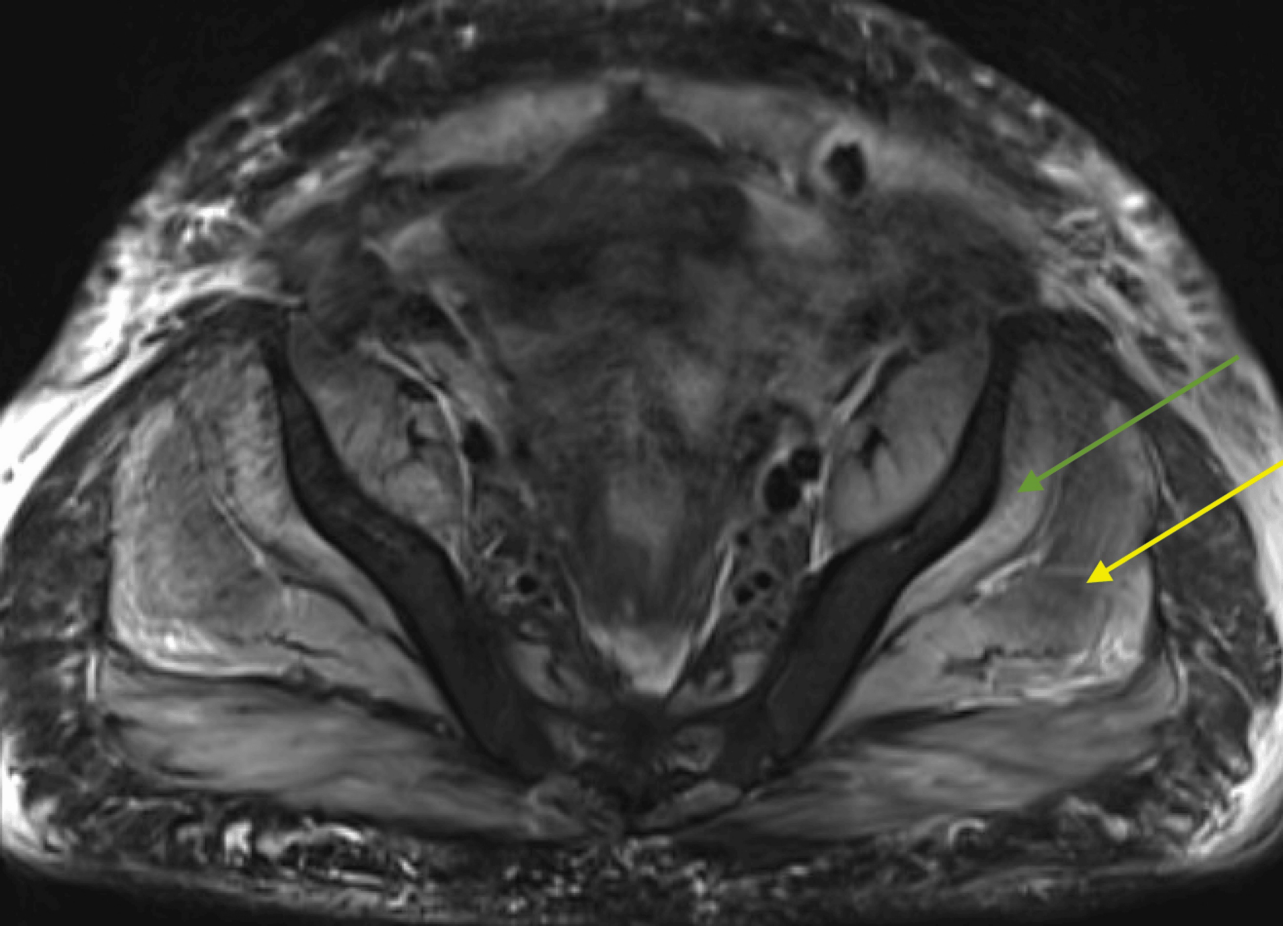
MRI of the left hip without contrast demonstrating muscle edema in the bilateral gluteus medius (yellow arrow) and gluteus minimus (green arrow).

Despite several days of fluid resuscitation, his creatinine continued to rise to 4.56 mg/dL and was accompanied by hyperkalemia with a serum potassium level of 5.7 mg/dL. The patient subsequently began to exhibit signs of volume overload as evidenced by oliguria without a robust response to intravenous diuretics. Internal jugular vein dialysis access was obtained one week into the admission, and dialysis was initiated. During the peri-dialysis period, the patient noted an exacerbation of muscle weakness, including the proximal upper and lower extremities. MRI of the left hip was performed and showed marked edema throughout the subcutaneous fat overlying the bilateral gluteus minimus and medius muscles without gross atrophy, as well as trochanteric bursal edema and mild ascites (Figure 3).

Muscle biopsy was declined due to severely elevated CPK levels and concern that the biopsy would have a low diagnostic yield given the degree of rhabdomyolysis. Neurology consultants opted to empirically treat the patient for statin-induced necrotizing myositis and initiated intravenous methylprednisolone 1 g daily for five days, followed by prednisone 1 mg/kg/day. Hydroxy-3-methyl-glutaryl-coenzyme A reductase (HMG-CoA reductase) inhibitor antibodies were obtained after initiation of pulse-dose steroids, and serologies returned negative.

CPK trended downward to 200 IU/L after three weeks on the inpatient service. The patient was ultimately discharged from the hospital to subacute rehabilitation with dialysis scheduled three times weekly and 60 mg of oral prednisone. HMG-CoA reductase inhibitor antibodies were repeated after hospital discharge and remained persistently negative.

## Discussion

The underlying pathophysiology of statin-induced necrotizing myositis is poorly understood, and several hypotheses have been proposed. HMG-CoA reductase is upregulated in a pre-disposed demographic due to chronic ingestion of statins, directly resulting in myotoxicity. Another proposed mechanism is a direct autoimmune-mediated process in which the body produces anti-HMG-CoA reductase antibodies, leading to a dysregulated immune response and targeting of muscle tissue, resulting in a rhabdomyolysis-like presentation. Genetic human leukocyte antigens associated with this disease process are HLA DRB1*11 and HLA DRB1*07 [2]. Note that rosuvastatin, pitavastatin, and atorvastatin account for approximately 80% of cases of statin-induced necrotizing myositis. Data on the exact doses and timing of initiation of these medications remain unclear, as this information was unknown in many reported cases [6].

Despite advancements in detection methods, from immunofluorescence to enzyme-linked immunosorbent assay (ELISA), HMG-CoA reductase inhibitor antibodies were negative. One possible explanation is that the patient had begun a course of stress-dose steroids before test collection, which may have limited the sensitivity of the test. Repeat antibody testing performed several weeks after hospital discharge also returned negative. At the time of the second test, the patient had completed the course of stress-dose steroids and was finishing an oral steroid taper. Nonetheless, detection of anti-HMGCR antibodies using ELISA has a sensitivity of 94.4% and a specificity of 99.3% [7]. Notably, the initial level of anti-HMGCR antibodies correlates positively with both serum CPK levels and clinical severity at presentation. The CPK level was 42 at the time of repeat testing, which likely explains the negative serologic result [7]. This highlights the importance of prompt testing in patients with suspected myopathy.

In patients with creatine kinase levels approximately ten times the upper limit of normal, it is recommended to pursue musculoskeletal imaging (e.g., MRI) or obtain electromyography (EMG) [8]. If anti-HMG-CoA reductase antibodies are positive, it is then recommended to proceed with a muscle biopsy. Although a muscle biopsy is not necessary to confirm the diagnosis, it can support the diagnosis if an unclear etiology persists despite antibody testing [8]. Negative antibody testing may help exclude statin-induced myopathy, and further evaluation for autoimmune or other causes of myositis is recommended [8]. In this patient, EMG was performed two months after hospital discharge and demonstrated a minimally abnormal study. 

Given the rapid clinical decline, the initial treatment plan included high-dose steroids due to concern for an unspecified myopathy. Treating patients with severe myopathy associated with rapid-onset muscle weakness or significant organ damage with pulse-dose steroids is consistent with the standard of care [9]. Subsequently, rheumatology consultants recommended a broad autoimmune workup, including ANA, dsDNA, C3/C4, a myositis panel, inflammatory markers, and immunoglobulin levels; however, no clear etiology was identified. Neurology consultants were initially concerned for myositis, and the preliminary diagnosis was documented as likely statin-induced necrotizing myositis.

Infectious disease consultants treated the patient empirically for Lyme disease-induced rhabdomyolysis, given low-positive VlsE1 IgM/IgG titers. The patient completed a 21-day course of doxycycline 100 mg every 12 hours. Repeat Lyme VlsE1 testing yielded negative titers one month after hospital discharge. anti-Jo-1, anti-Scl-70 IgG, anti-centromere B, anti-ribosomal protein, anti-MPO IgG, anti-PR3 IgG, anti-glomerular basement membrane antibody, anti-dsDNA, anti-ENA Ro, anti-ENA La, anti-ENA Sm, and anti-ENA RNP titers were negative.

Several differential diagnoses were considered in this case, including polymyositis, inclusion body myositis, dermatomyositis, unspecified autoimmune myositis, and tick borne induced rhabdomyolysis. Lyme-associated myositis was considered unlikely by infectious disease consultants based on low positive Lyme titers on admission and the absence of a febrile prodrome consistent with Lyme disease. Polymyositis and dermatomyositis serologies drawn on admission were negative. Inclusion body myositis is typically associated with a 10- to 20-fold elevation in creatine kinase and most commonly manifests with weakness of the finger flexors and knee extensors, which was inconsistent with this patient’s presentation [10]. Exertional rhabdomyolysis was considered unlikely due to the absence of recent physical activity or traumatic injury noted on admission. Drug-related rhabdomyolysis was also considered less likely given a negative urine drug screen on admission and the absence of relevant exposures in the presenting history. Several factors supported the diagnosis of statin-induced necrotizing myositis over the aforementioned conditions, including older age, male sex, lack of autoimmune history, no history of past or active malignancy, and the use of a high intensity statin. Notably, the patient was also taking amlodipine, which has been associated with increased atorvastatin levels through CYP3A4 inhibition and may have contributed to the development of this presentation [11].

## Conclusions

This case highlights the clinical features, workup, and differential diagnosis of suspected statin-induced necrotizing myositis. Establishing the most probable diagnosis was challenging in this case, as laboratory markers were insufficient and additional testing, including EMG, was likely falsely negative because testing was performed after significant clinical improvement had already occurred. Diagnosis, therefore, required a comprehensive medical evaluation, which included a thorough medication reconciliation, identification of potential risk factors or lack thereof on presentation, along with the overall clinical picture and multidisciplinary discussion among several consultants, including neurology, rheumatology, and nephrology. There are no prophylactic interventions that providers can implement to predict or prevent a fulminant myositis, and inpatient and outpatient providers alike should remain vigilant of this rare and debilitating diagnosis. 
